# Advancements in Tissue-Equivalent Gel Dosimeters

**DOI:** 10.3390/gels11020081

**Published:** 2025-01-21

**Authors:** Mustafa Erdem Sagsoz, Ozlem Korkut, Salvatore Gallo

**Affiliations:** 1 Department of Biophysics, Faculty of Medicine, Atatürk University, 25050 Erzurum, Türkiye; 2 Department of Chemical Engineering, Faculty of Engineering, Atatürk University, 25050 Erzurum, Türkiye; 3 Department of Physics and Astronomy “Ettore Majorana”, Catania University, via Santa Sofia 64, 95123 Catania, Italy; salvatore.gallo@unict.it

**Keywords:** gel dosimeters, tissue equivalency, radiotherapy, three-dimensional dosimetry, optical density, magnetic resonance imaging

## Abstract

Tissue-equivalent hydrogel dosimeters represent a class of tools that hold significant promise, particularly in the precise measurement of three-dimensional dose distributions in radiotherapy. Due to their physical properties closely resembling those of human soft tissue, these dosimeters effectively replicate the energy transfer phenomena resulting from radiation interactions, such as atomic ionization and scattering by nuclei or electrons. Consequently, tissue-equivalent dosimeters, characterized by their linear energy transfer properties, have been extensively applied in medical physics, radiation oncology, and nuclear safety. Future advancements focusing on developing more stable, less toxic, normoxic, and cost-effective dosimeters could enable their broader adoption. This review provides a comprehensive overview of the key characteristics that make hydrogel dosimeters tissue-equivalent, highlighting their benefits, limitations, and primary application areas. Additionally, it explores current advancements in polymeric gel technology and discusses future directions aimed at optimizing their performance and accessibility for broader adoption.

## 1. Introduction

Radiotherapy continues to evolve as a discipline where precise and personalized treatment planning is increasingly critical. In this context, the accurate and reliable application of radiation dosimetry techniques is essential for enhancing treatment efficacy and protecting surrounding tissues.

Radiation dosimetry in the fields of health physics and radiation protection is the measurement, calculation, and assessment of the ionizing radiation dose absorbed by the human body. A major challenge in radiotherapy treatment is to deliver a precise dose of radiation to the tumor with a minimum dose to healthy normal tissues. Radiation measurements cover a broad area of instruments and methods focusing on measurements of different parameters of radiation.

Validation and radiation process control depend on the measurement of the absorbed dose, which should be performed using a dosimeter. The dosimeter is a device that measures direct or indirect exposure, kerma, absorbed dose, equivalent dose, and other related quantities.

Kerma (kinetic energy released to matter, *K*) measures the amount of energy that is transferred from photons to electrons per unit mass at a certain position (1).

(1) $$K=\frac{d{\bar{E}}_{tr}}{dm}$$

K is related to but not the same as the absorbed dose (D), which measures the energy deposited in a unit mass at a certain position (2).

(2) $$D=\frac{d{\bar{E}}_{ass}}{dm}$$

Both K and D are measured by Gray (J·kg^−1^). Finally, the equivalent dose (HT), is a measure of the radiation dose to tissue and represents the health effect of a low level of ionizing radiation on the human body. The unit of measure is Sievert (J·kg^−1^), and it is calculated by multiplying the absorbed dose to the organ or tissue (*D_T_*) with the radiation weighting factor, WR. This factor is dependent on the type and energy of the incident radiation (3).

(3) $$H_{T}=\sum_{R}W_{R}{\times D}_{T}$$

The dosimeter, along with its reader, is referred to as a dosimetry system. To be useful, the dosimetry system must exhibit several properties such as high accuracy and precision, linearity, dose or dose rate dependence, energy response, directional dependence, spatial resolution, and radiological tissue equivalence [1].

Among the various dosimetry systems available, gel dosimeters are particularly notable; these systems typically consist of chemicals embedded within an aqueous gel matrix that are activated by radiation [1].

Dosimeters designed to minimize the effects of radiation doses on non-target tissues during radiotherapy and to map the beam distribution within target and adjacent tissues often prioritize the use of phantoms. Experimental setups for dosimetry simulations are widely employed to replicate the interaction of radiation with tissue. These setups include homogeneous water phantoms, water-equivalent solid phantoms, and heterogeneous phantoms made from materials such as wax, plastic, and acrylic. These materials are designed to mimic the radiation interaction characteristics of various human tissues [2]. However, as these phantoms are generally non-disposable, assessing changes in tissue equivalence due to radiation exposure remains challenging.

In a study where gel dosimeters were used to evaluate the dose enhancement potential of gold nanoparticles (AuNPs) in radiotherapy, it was shown that AuNPs provide dose enhancement by increasing photon interactions due to their high atomic number [3].

Gel dosimeters provide a 3D measurement of dose distribution in patient-specific quality assurance (QA) studies in radiotherapy, which is an important advantage in the verification of complex treatment plans [4,5]. Polymer gel dosimeters provide high spatial resolution, especially in small target volumes and high dose gradients [6,7]. In studies on the accuracy and reliability of gel dosimeters, it is stated that the dose–response curve is generally linear and repeatable, which makes them preferred in procedures requiring precision [8,9]. Gel dosimeters can be used with magnetic resonance imaging (MRI), which is suitable for dosimetry in MR-Linac systems [10,11]. Gel dosimeters have different application areas in complex treatment modes, such as stereotactic radiosurgery (SRS), IMRT, and SBRT [6,7]. They can also be used for the fast determination of Linac isocenter [12]. The disadvantages of gel dosimeters are that they are sensitive to changes in ambient temperature, and this can affect the measurement results [8]. The preparation and analysis of measurements are time-consuming [5,13]. In low-dose regions, inhomogeneous structures at the air/gel interface may cause deviations in the results [4,8]. On the other hand, calibration may be required before each use, which can complicate the experimental design [6]. In addition, the production and supply of gel dosimeters can be more costly compared to other dosimetry methods [5,7]. The use of gel dosimetry with other methods (e.g., film dosimetry) can increase measurement accuracy and reliability [10,11].

Superficial energy transfer and a high dose rate for electron beams, especially with FLASH radiotherapy [14], can also be characterized with gel dosimeters. For example, the MR R2-dose response and the sensitivity of the PASSAG gel dosimeter were shown to be similar for electron beam energies compared to a photon beam (the differences were less than 5%) [15].

Gel dosimeters, in contrast, offer high adaptability and are particularly suitable for three-dimensional dose measurements. They can be classified into several categories based on their composition, including Fricke gel, radio-chromic, gellan gum, hydrogel, and polymer gel dosimeters [16]. While the classification of gel dosimeters varies in the literature, they can broadly be categorized according to their chemical content, encompassing multiple existing classification systems.

## 2. Gel Dosimeters

### 2.1. Fricke Gel Dosimeters and Hydrogels

Fricke gels, first developed in the 1920s, have played a foundational role in advancing dosimetry technologies through chemical, physical, and imaging-based analysis methods. These gels are dosimeters that utilize the radiation-induced conversion of ferrous ions (Fe^2+^) into ferric ions (Fe^3+^) [1]. The amount of ferric ion produced is directly proportional to the absorbed radiation dose. This makes Fricke gels effective as dosimeters for measuring the spatial distribution of radiation. The transformation induces notable changes in the magnetic properties of the gel, altering spin–spin and spin–lattice relaxation times. Such changes enable the precise detection of radiation doses using magnetic resonance imaging (MRI) and various optical techniques.

One of the primary advantages of Fricke gels is their well-characterized chemical behavior. Furthermore, they exhibit properties like soft tissue, making them highly suitable for medical and biological applications. However, there are notable limitations: they are effective only within a dose range of 10–40 Gy, and their accurate imaging requires rapid processing—preferably within two hours post-irradiation—due to the high diffusion rate of Fe^3+^ ions [17].

To address these challenges, extensive research has been conducted on improving Fricke gels. Efforts include incorporating chelating agents, such as xylenol orange and methyl thymol blue, to reduce ion diffusion. Similarly, additives like polyvinyl alcohol (PVA), glutaraldehyde, and nanogels have been explored to enhance gel stability and mitigate diffusion issues [16,18]. For instance, studies by Penev and Mequanint [19] demonstrated that rheology modifiers significantly improve the structural integrity and stability of Fricke gels.

Innovations have also aimed at mimicking human tissue properties more closely. Dosimetric Fricke gel formulations enriched with additives like calcium chloride (CaCl_2_) or gluconic acid have shown improved similarity to human tissues in terms of X-ray interactions [20,21]. In another comparative study, two gel dosimeters made from agar and bovine gelatin were evaluated under similar production conditions. Magnetic resonance (MR) T1-imaging responses revealed a clear dose dependence of MR intensities. The findings indicate that agar-based Fricke gels produce more consistent and reliable dosimetric results compared to gelatin-based gels [22].

These advancements highlight the potential of Fricke gels as versatile tools in radiation dosimetry, paving the way for improved accuracy in therapeutic and diagnostic applications. Further research into enhancing their stability, extending their operational dose range, and refining imaging techniques will undoubtedly expand their utility in clinical and experimental settings.

### 2.2. Polymer Dosimeters

In these types of dosimeters, radiation-induced polymerization occurs. Some of these that are sensitive to oxygen are polyacrylamide gels PAG or PAC (containing acrylamide + acrylic acid), BANG (methylene bis acrylamide as crosslinker + acrylamide as monomer + gelatin), MAG (methacrylic acid as monomer), and NIPAM (less toxic n-isopropyl acrylamide). The polymerization of these gels is slowed down by oxygen. While MAG, PAG, and VIPET use THPC as an antioxidant, MAGADIT contains dithiothreitol to reduce disulfide bonds [23]. Cinq-Mars and his team compared two different NIPAM-based gel recipes and emphasized the advantages of low-concentration NIPAM gels in terms of economy and sensitivity [24]. Those referred to as normoxic polymer gels can be produced under normal oxygen-containing atmospheric conditions. VIPET (normoxic N-vinylpyrrolidone-based gel) [25] and MAGIC (containing methacrylic acid, gelatin, ascorbic acid, copper II sulfate, and hydroquinone) are examples. Farhood et al. developed a low-toxicity polymer gel containing AMPS monomer instead of acrylamide. PASSAG stands out with its environmental friendliness, low toxicity, water equivalence, and linear dose response [26].

In some studies where gel dosimeters were classified, radiochromic polymer gels were examined under the title of polymer gel dosimeters [17]. These gels are hydrogels whose optical transmittance at certain wavelengths changes with irradiation. GENIPIN, leuco crystal violet (LCV), and Tetrazolium Gellan Gum Gel are common examples [27]. Micellar gel dosimeters consisting of a radiation-sensitive hydrophobic dye (leucomalachite green), an organic halogen (chloroform) as an initiator, and a surfactant (sodium dudosyl sulfate, SDS) for stabilization are also radiochromic gel dosimeters. Similarly, TruViewTM, where methyl thymol blue (MTB) is added to commercially available Fricke gel as an indicator, and Clear ViewTM, where tetrazolium salts are converted into insoluble formazan dyes when exposed to radiation, can be counted in this group. Jaszczak-Kuligowska and her team designed an elastic gel containing nitro blue tetrazolium (NBT) and showed that this gel can be used both as a bolus and as a dosimeter [28]. Penev and Mequanint proposed a new formulation based on tetrazolium salts to improve dose response and reduce water loss [19]. In addition, PVA–iodide gels are radiochromic gels based on the formation of tri-iodide ions from the reaction of the chemical structures released by the water in their content because of irradiation with the iodine in the environment, and then these ions form a complex with PVA [16]. The polyvinyl alcohol and iodine complex (PVA-I) developed by Hayashi and his team offers many advantages, such as high sensitivity, water equivalence, a wide dose range, and reusability. In addition, it has been stated that this gel is suitable for optical tomography scanning-based 3D dosimetry [1,16].

**Figure 1:**
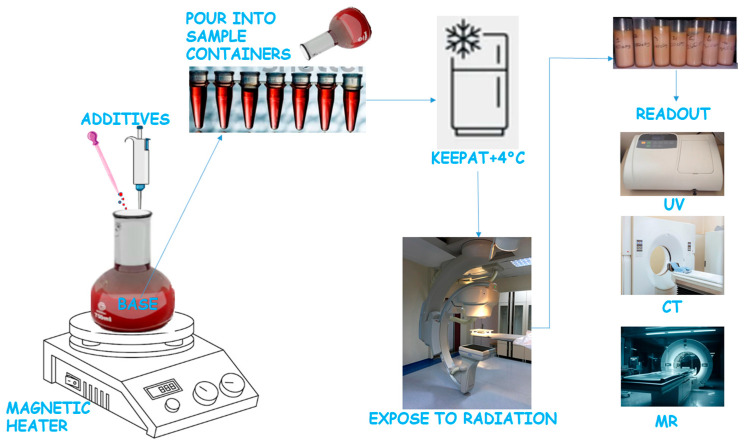
Fabrication, exposure, and readout procedures for gel dosimeters.

A general view of fabrication, exposure, and readout procedures for gel dosimeters is shown in Figure 1.

### 2.3. Solid Plastic Dosimeters

These dosimeters operate on the principle of radiation-induced polymerization of plastic materials, making them highly effective for certain types of radiation dosimetry. A notable example is the PRESAGE gel, which consists of a dye embedded within a plastic matrix composed of polyurethane and silicone. This innovative design ensures stability and usability in three-dimensional dose measurement applications [29,30].

Another example is the Flexydos3D dosimeter [31,32], which features an elastomer-based matrix combined with halogens and leucodyes as initiators for polymerization. The unique composition of Flexydos3D enhances its flexibility and adaptability for varied radiation environments. When examining their composition and mechanism of action, these dosimeters can be classified as radio-chromic gel dosimeters. The term “radio-chromic” refers to their ability to undergo a measurable color change upon exposure to radiation. This characteristic enables the precise visualization and quantification of radiation dose distribution. These dosimeters provide significant advantages in applications requiring detailed 3D imaging and are widely valued for their stability, accuracy, and ease of use in modern dosimetry [17,18].

### 2.4. Radiofluoregenic or Radiophotoluminescence Dosimeters

Radiophotoluminescence (RPL) is usually seen in single crystals, glasses, and ceramics. However, it is known that soft materials such as liquids, gels, and organic solids can also be fluorescent after irradiation [33].

Examples of liquid solutions used in chemical fluorescence dosimetry that perform RPL include aqueous coumarin solutions (coumarin-3-carboxylic acid solution), aqueous benzoic acid solutions, terephthalic acid solutions, and trimesic acid. Up to 1 millimolar of gold nanoparticles have been added to aqueous coumarin solutions to increase sensitivity [33]. Dosimeters produced in gel form and performing RPL are included in the group of radio-fluoregenic gel dosimeters. The main operating principle depends on the production of fluorescent molecules by a chemical reaction with OH radicals. An example of these gels is the gel obtained by adding MPy solution to the structure formed by the partial polymerization of tBuA with gamma rays. Nanoclay, rhodamine 123 (RD 123), DHR 123, halogen, and Fe^3+^ ions as catalysts and pyridine as dispersants were added to these gels to improve their properties [16]. The addition of methyl methacrylate to gels containing MPy results in copolymerization. This type of gel can also be produced by adding agarose, gelatin, and nano-clay to coumarin-3-carboxylic acid solutions [16].

Solid-phase organic dosimeters can also exhibit luminescence after irradiation. Fluorescence is obtained as a result of the chemical reaction of radicals formed from the main component with a dye after irradiation. For example, the presence of pararosaniline leuco dye and polyethylene glycol diacrylate in the polymer-based solid can achieve this [34]. Gel dosimetry studies have focused on aspects such as increasing sensitivity, reducing toxicity, and providing ease of use. Changes in polymer compositions (e.g., NHMA and PASSAG) [35] overcome toxicological problems, while new matrix designs have wider application areas and allow for reusability (e.g., Pluronic F-127) [36].

## 3. Tissue Equivalence

Tissue equivalence refers to the property of a material or substance that mimics the physical and biological characteristics of human tissue when exposed to ionizing radiation. This means that the material interacts with radiation in a similar way to human tissues in terms of both energy absorption and scattering. Tissue equivalence is important in dosimetry because it allows for accurate measurements of the radiation dose in models or phantoms that simulate the human body, thereby improving the accuracy of radiation therapy and diagnostic procedures. In the case of dosimetric hydrogels, materials with tissue equivalence can be used to replicate how radiation will behave within the human body, allowing for more precise calculations of radiation doses delivered to detailed areas.

Treatment planning systems (TPSs) using the Monte Carlo algorithm can calculate the dose (D) delivered to tissues that are not water-equivalent, such as bone, using various coefficients. There are some algorithms that can calculate the build-up dose in different tissue-equivalent materials [37]. However, these values cannot be verified using real measurements. To determine these, it is necessary to develop tissue-equivalent materials or, better yet, tissue-equivalent dosimeters [38]. In addition to the density of the dosimetric material, it is important to find the approximate chemical formula, even if the effective atomic number and chemical bonding effects are ignored in determining tissue equivalence. Table 1 shows the approximate chemical formulas calculated based on the density and atomic weight fractions of the dosimetric material, last updated in 2004, based on the International Commission on Radiation Units & Measurements—ICRU 44 [39,40] report by Hubbel and Seltzer.

Table 1 and Table 2 also include variables indicating the tissue equivalences of various dosimetric materials, such as the density and effective atomic number.

**Figure 2:**
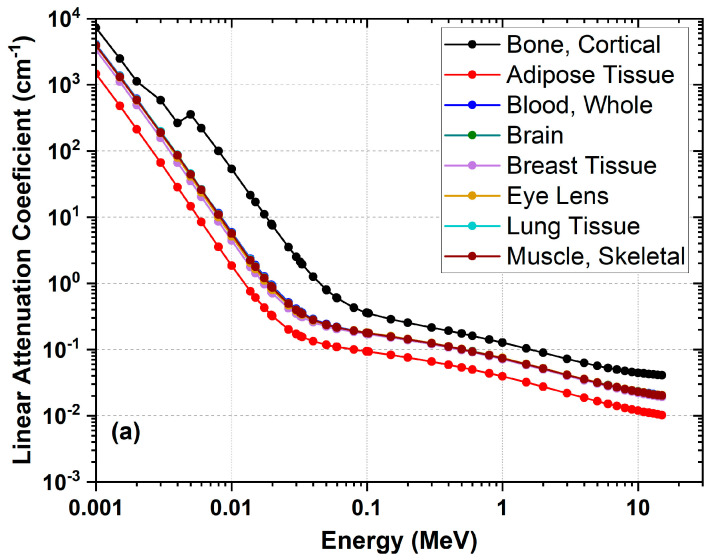

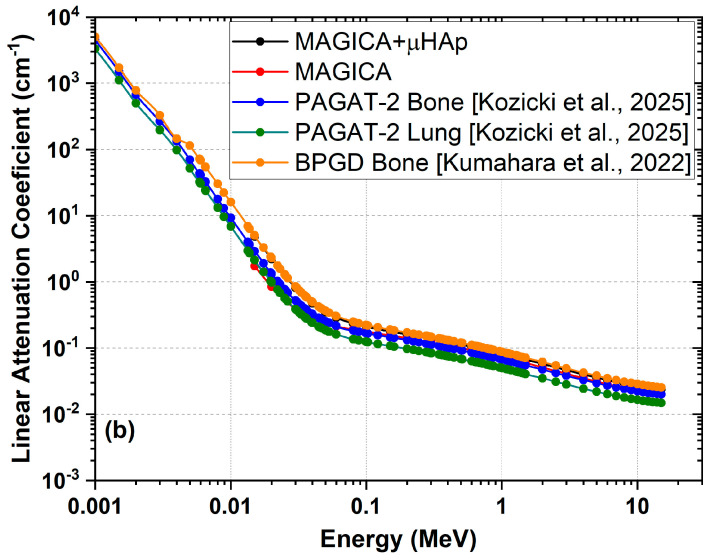
Linear attenuation coefficients (LACs) of human tissues (**a**) and common hydrogel dosimeters (**b**) ranging between diagnostic and therapeutic X-ray energies. Data taken from [41,42].

High sensitivity and accuracy in radiotherapy are critical, especially in the treatment planning of complex tissues. Bone-like polymer gel dosimeters offer an innovative solution in this field due to their ability to measure three-dimensional dose distribution and their tissue-mimicking properties. One of the most important variables to mimic tissues in terms of physical interaction is to have similar linear attenuation coefficients to real tissues at diagnostic or therapeutic X-ray energies (see Figure 2).

Traditionally, water-mimicking dosimeters have been widely used; however, the development of gel dosimeters that mimic different types of tissues allows for the validation of more complex treatment planning setups. For example, polymer gels such as PAGAT2–Pluronic F–127 can mimic the absorption properties of bone tissue with the addition of components such as calcium hydroxyapatite [43,44]. The effect of different hydroxyapatite concentrations to simulate bone trabecular structure and dense bone properties has also been investigated in detail [42,45] (Table 2).

Also, the addition of MgCl_2_ to systems such as BoneGel can increase the sensitivity of the dosimeter, thus improving the accuracy of radiotherapy plans [45]. The developed systems not only mimic different tissue types, but also allow for the measurement of complex dose distributions on a whole-body model by creating multiphase dosimeters. In recent years, the use of hydrogels in dosimetry systems has attracted great interest. These gels are preferred not only for their tissue-mimicking properties, but also for their capacity to measure dose distributions with three-dimensional geometry [43]. In particular, the use of cross linkers and stabilizers in hydrogel structures to increase stability and improve sensitivity is noteworthy [46].

**Table 2:** Physical properties of different gel dosimeters.

Dosimeter Type	Samples	Approximate Chemical Formula *	ρ [g/cm^3^]	<Z/A>	Zeff
Polymer Gel Dosimeters	PAGAT, MAGAT, NIPAM	C15H16O116N3PS1Cl1K1 MgCl doped PAGAT2 [44]	1.12	0.542	10.71
Radiochromic Polymer Gels	PRESAGE	C_29_H_57_O_14_N_9_S [47]	1.05	0.540	7.3
Solid Plastic Dosimeters	PMMA	C_5_H_8_O_2_	1.190	0.539	6.47
Radiophotoluminescence	RPLDs	C_596_H_741038_O_370212_N_156_Na_3_P_1_ [48]	2.20	0.500	12.00
Solid Phase Organic Dosimeters	TLDs	LiF	2.635	0.463	3.92

* Complex approximate chemical formulas derived from corresponding references.

## 4. Measurement Procedures

One significant topic in tissue-equivalent dosimetry is the selection of methods for measuring the gel’s response to the applied dose and determining the dose distribution. Various reading techniques, such as MRI, CT [49], optical CT [50], and ultrasonic imaging, have been employed as readout systems [17].

A study published by Simon J. Doran in 2019 delved into the fundamental principles and technological advancements of 3D dosimetry reading techniques. Doran provided a comparative analysis of these methods, highlighting their critical role in radiotherapy processes [51].

### 4.1. NMR and MRI Scanning Methods

Magnetic resonance (MR) readout methods, unlike the optical scanning methods of gel dosimeters, obtain dosimetry data by using magnetic fields and the magnetic properties of protons. In MR-based methods, the chemical structure of the gel material, especially the environmental changes around the protons, has a great effect on the measurements. MR readout methods are sensitive to various properties of the materials contributing to the gel dosimeter.

Polymer gel dosimeters are hydrogels containing vinyl monomers and usually a crosslinker monomer. When exposed to ionizing radiation, radiation-induced water radicals will initiate a polymerization reaction, resulting in the formation of small polymer aggregates surrounded by the hydrogel matrix. This sudden change in polymerization influences the NMR transverse relaxation rate, which can be used to obtain quantitative R2 maps related to the absorbed dose [52].

The magnetic resonance properties of gel dosimeters may depend on different mechanisms. Proton density and distribution are important, the water content should be high, and T1 and T2 spin dynamics can change with dose. The polymeric structure, water content, and crosslinking ratio affect T1 and T2 relaxation times. Free radicals generated by radiation may change the MR signal. A gelatin or polymer chemical structure creates radicals. Water–polymer interactions change with an increasing dose. The strength and formation time of the polymer network change T1 and T2. Saturated and viscous structures increase the accuracy of the MR signal. For this reason, gels with a relatively high-water content are more sensitive in terms of MR scanning [53]. The binding capacity of water to gel polymers also affects MR data [54]. Polymerization, water content, and crosslinks formed by the effect of radiation affect the T1 and T2 relaxation times of protons [55]. Free radical formation, one of the most important results of radiation–matter interaction, also leads to changes in MR parameters (for example, T2 time) [56]. The change in the molecular order in the gel by the crosslinking rate and polymerization time affects the movement of water and the magnetic properties of protons. Therefore, it changes the T1 and T2 times [57]. Solvents and additives in the gel content can affect the solubility and saturation properties. The solubility determines the homogeneity and signal strength of the gel in MR relaxation studies. The resolution ratio affects the precision of the MR signal of the gel. Saturated or viscous structures allow the protons to spread more homogeneously and increase the accuracy of the MR signal [58].

Rabaeh and his team developed a new polymer gel based on NHMA. This gel is suitable for both MRI and optical tomography-based dosimetry due to its stable performance under different irradiation conditions [59]. De Deene and Mason developed gadobutrol-doped polymer gels optimized for MRI-based real-time dosimetry [49].

Chemical stability is an important factor in the post-irradiation MR parameters of the gel. Unstable chemicals can degrade rapidly in proportion to the dose, which can reduce the accuracy of the measurements. Chemical stability is particularly related to the monomers or crosslinkers used. Unstable compounds can create erroneous readings in the MR signal that are not related to the radiation intensity [60].

MR readout methods are particularly sensitive to proton density, spin dynamics, radical formation, and chemical changes during the dose measurement of gel dosimeters. Therefore, the content, chemical structure, and physical properties of the gel material used can directly affect the accuracy and sensitivity of MR techniques. A better understanding of these interactions could encourage new material designs for more sensitive and accurate dosimetry.

### 4.2. X-Ray CT Scanning

X-ray CT (Computed Tomography) readout methods are sensitive to certain material properties that are important for the dose measurement of gel dosimeters and for achieving tissue equivalence. These properties are generally related to how the gel material will affect the X-ray beam. This process depends on factors such as the density, atomic number, water content, and chemical structure of the material. X-ray CT technology relies on the analysis of these properties to provide accurate measurements for dosimetry [22,61,62]. Heavy elements added to the gel material, such as iodine or barium-based compounds, increase X-ray absorption. This allows dosimeters to provide higher X-ray contrast. Additives with higher densities and atomic numbers can improve tissue equivalence [63]. A high-water content can be useful in simulating the interaction of X-rays with the human body, so as the water content increases, dosimeters become more suitable, especially in terms of soft tissue equivalence [64]. The chemical content similarity of organic compounds in polymer structures to body tissues can increase the accuracy in X-ray CT scans [65]. Ensuring the bioequivalence of the gel material relies on the appropriate selection of compounds that interact with X-rays [66].

With the use of rapid dose response and room-temperature-stable gel materials, new CT-based dosimeters have also been developed [67].

For materials suitable for these measurement methods to provide bioequivalence and tissue compatibility, they must have high densities and atomic numbers; correctly adjusted water contents; contain carbon-, hydrogen-, oxygen-, and nitrogen-like body tissue; have chemical similarity to organic structures; and have a high crosslinking ratio.

### 4.3. Optical Scanning

Methods such as UV-Vis spectroscopy and spectrophotometry are sensitive to changes in optical absorption caused by radiation in gel dosimeters. Additives added to the gel (e.g., leuco-dye or metal complexes) produce significant changes in the absorption spectrum when exposed to radiation. Of these changes, wavelength-dependent absorption and the absorption maxima of additives are measured by UV-Vis absorption spectroscopy and are related to radiation dose. The increase in the absorption coefficient and chemical products formed because of radio-chromic reactions increase the absorption intensity at different wavelengths. The sensitive substances are metal chelates such as xylenol orange, iron ions in Fricke solution (Fe^2+^/Fe^3+^ conversion), and radio-chromic dyes (e.g., nitrophenol derivatives). The compatibility of various gel types, such as Fricke-Xylenol Orange-Gelatin (FXG), PAKAG, and NHMA, with optical scanning methods and their performance in different radiotherapy scenarios have been compared. The dual-wavelength optical CT scanning method provides high sensitivity. In the study by Rousseau et al., dose measurements obtained with this method showed high agreement between 90 and 40% in isodose curves and a pass rate between 96.7 and 98.6% with a gamma analysis. This method provides high accuracy even in out-of-field regions, especially in volumetric modulated arc therapy (VMAT) plans [68,69].

The PAKAG gel provides consistent results with UV-Vis. Abtahi and Habibi studied the optical response of PAKAG gel by UV-Vis spectroscopy. The study showed that the response of the PAKAG gel is stable over a wide dose rate range and gives consistent results at different energy levels [69]. Some dosimeters use substances such as radiosensitive fluorescent radio-chromic fluorescent dyes or organic phosphor compounds. The emission intensity or spectral position of fluorescent substances changes under the influence of radiation. Optical methods can determine the radiation dose by measuring these changes. For example, fluorescence intensity is usually directly proportional to the radiation dose. Stokes shift is the shift in the emission spectrum because of chemical changes due to radiation [70].

Optical tomography methods are sensitive to physical changes in the gel dosimeter caused by radiation, such as volumetric shrinkage, density increase, and organic solvents, crosslinkers, and stabilizers used for the gel matrix [71].

In complex techniques such as craniospinal irradiation (CSI), optical CT-based measurements are critical tools for assessing the dose accuracy at field intersections. The work of Silveira et al. showed a high success rate of 96.91% with a gamma analysis [72].

Raman spectroscopy is used to analyze chemical structure changes in radio-chromic reactions. Raman spectroscopy is sensitive to radiation-induced molecular structure changes in gel dosimeters. New bonds formed in molecules because of radiation or existing bonds broken are detected as characteristic vibration bands in the Raman spectrum. For example, in polymerization reactions in acrylamide-based gel dosimeters, C=C bonds formed during radiation polymerization are depleted or new C-C bonds are formed. The structure of ionic additives changes under the influence of radiation. Examples of sensitive substances are acrylamide monomers that facilitate polymerization, such as chemical crosslinkers (e.g., bis-acrylamide).

The study by Chacón et al. showed that Raman spectroscopy is an effective tool for the detailed analysis of polymeric structures used in dosimeters [73]. Although Raman spectroscopy provides high resolution, it generally requires more expensive equipment and is therefore less widely used.

Volumetric scanning methods such as laser CT are sensitive to changes in optical density and refractive index caused by radiation. Loss of homogeneity or local density changes occur in the gel dosimeter under the influence of radiation, which affects the refractive properties of light. In particular, the refractive index gradient represents the optical density distribution in the gel, while the increase in optical density is a result of polymerization or crosslinking reactions.

Optical scanning methods become powerful tools in radiotherapy QA applications when combined with different types of gels. In the future, it is expected that the sensitivity of these techniques will be increased, and they will be used in a wider range of applications.

Other scanning techniques generally include tomography-based or photonic detection methods. One example is CBCT (Iterative Cone Beam Computed Tomography). Kozicki et al. tested the sensitivity of 3D polymer gel dosimeters with iCBCT technology. This method has been used in the verification of complex radiation fields and isocenter tests [74].

Among optical signal-based approaches, Yang et al. examined different optical signal responses in passive dosimeters and showed that such signals can be used in a wide range of applications [75].

In addition, Colnot et al. presented a unique quality assurance method for radiotherapy by combining gel dosimetry with 3D-printed personalized phantoms [76].

Each of these techniques offers important contributions for radiotherapy QA applications. However, the method of choice may vary depending on the application. For example, UV-Vis and spectrophotometry are less costly and applicable methods, while laser CT and Raman spectroscopy provide higher resolution and accuracy. Volumetric measurement methods, such as laser CT, are preferred for analyzing complex dose distributions, while UV-Vis spectroscopy is generally suitable for simpler dosimeter analyses. In addition, Raman spectroscopy has an important place in chemical analyses because it can provide information at the molecular level.

In Table 3, the basic structures of common dosimetric gels and the relevant measurement techniques after irradiation are given [3,20,43]. The raw materials expressed as bases and other chemicals as additives are given for tissue-equivalent gel production.

## 5. Conclusions

In conclusion, research involving personalized phantoms significantly enhances the potential for patient-specific dosimetry, a critical aspect of modern medical radiation applications. These studies pave the way for more accurate and individualized radiation therapy, enabling the precise delivery of doses tailored to the unique anatomical and physiological characteristics of each patient.

Looking ahead, future optimization efforts are expected to concentrate on the development of versatile, multipurpose gel dosimeters that can adapt to various radiation types and dose rates. Such advancements will further broaden the applicability of dosimetric gels in diverse clinical settings, enhancing their utility in both diagnostic and therapeutic radiation procedures.

In the realm of patient-specific radiotherapy planning, particularly in cancer treatments, the use of dosimetric gels proves to be invaluable. These gels offer a reliable method for delivering radiation precisely, minimizing the risk of damage to surrounding healthy tissues. Many researchers who have contributed to this field have recently focused on improving existing gel formulations, such as Fricke gels, polymer gels, and solid plastic gels. They have incorporated a range of additives to better simulate the characteristics of human tissues, ensuring more accurate dose delivery and tissue equivalence [77,78,79,80,81,82,83].

Furthermore, they have developed appropriate measurement methods to enhance the precision and reliability of these dosimetric tools [84].

Ultimately, these advancements in dosimetric hydrogel technology are expected to play a pivotal role in the ongoing improvement of cancer treatment, enabling safer and more effective radiation therapy that is personalized for each patient’s unique needs. Personalized dosimetric studies will need different tissue-equivalent gel dosimeters to be optimized for each application. MR-Linac facilities may accelerate Fricke gel dosimeters, polymer dosimeters, and radio-chromic polymer dosimeters to be studied more effectively. Also, gel dosimeters can be adapted to mechanical phantoms mimicking respiratory movement. The advantages of radio-chromic polymer gels will be used for 4D dosimetry.

## Figures and Tables

**Table 1:** Approximate chemical formulas and physical properties of tissues and various dosimetric materials [39,40].

Material	Approximate Chemical Formula	ρ [g/cm^3^]	<Z/A>	Z_eff_
Adipose Tissue	C_1143_H_2594_N_11_O_401_Na_1_S_1_Cl_1_	0.555	0.554	3.27
Blood, Whole	C_212_H_2322_N_54_O_1071_Na_1_S_1_Cl_1_Fe_1_K_1_	1.060	0.550	3.66
Bone, Cortical	C_297_H_959_N_169_O_624_Na_1_Mg_2_S_74_Ca_129_	1.920	0.515	5.08
Brain	C_195_H_1724_N_25_O_718_Na_1_P_2_S_1_Cl_1_K_1_	1.040	0.552	3.62
Breast Tissue	C_1111_H_4249_N_86_O_1322_Na_2_Mg_2_S_3_Ca_1_	1.020	0.552	3.48
Eye Lens	C_576_H_3375_ N_144_O_1433_Na_2_P_1_S_3_Cl_1_	1.070	0.547	3.58
Ferrous Sulfate Standard Fricke	FeSO_4_·7H_2_O	1.024	0.553	5.61
Gadolinium Oxysulfide	Gd_2_O_2_S_10_	7.440	0.423	11.92
Gafchromic Sensor	C_7_HNO_2_	1.300	0.544	3.47
Lithium Tetraborate	Li_2_B_4_O_7_	2.440	0.485	3.64
Lung Tissue	C_171_H_1996_N_43_O_914_Na_2_P_1_S_2_Cl_2_K_1_	1.050	0.550	3.66
Muscle, Skeletal	C_425_H_3615_N_87_O_1585_Na_2_P_2_S_3_Cl_1_K_4_	1.050	0.550	3.64
Ovary	C_152_H_2043_ N_34_O_941_Na_2_P_1_S_1_Cl_1_K_1_	1.050	0.551	3.65
Polystyrene	C_8_H_8_	1.060	0.538	5.7
Polytetrafluoroethylene (Teflon)	C_2_F_4_	2.250	0.480	8.43
Polyvinyl Chloride	C_2_H_3_Cl	1.406	0.512	13.86
Radiochromic Dye Film	C_9_H_16_N_1_O_2_	1.080	0.550	6.2
Testis	C_15_H_16_O_116_N_3_P_1_S_1_Cl_1_K_1_	1.040	0.552	9.02
Tissue, Soft	C_23_H_34_O_119_N_5_P_1_S_1_Cl_1_K_1_	1.060	0.550	8.85
Tissue, Four-Component	C_6_H_13_NO_7_	1.000	0.550	7.02
Water, Liquid	H_2_O	1.000	0.555	7.42

**Table 3:** The basic structures of common dosimetric gels and relevant measurement techniques after irradiation.

	Fricke Gel Dosimeters	Polymer Dosimeters	Radiochromic Polymer Dosimeters	Solid Plastic Dosimeters	RPL Dosimeters
Bases	Water, Gelatine, Fe^2+^	Water, Gelatine, Monomer	Water, Gelatine, Surfactant/PVA	Plastic/Elastomer	Solutions ff Coumarin/Aqueous Benzoic Acid/Terephthalic Acid/Trimesic Acid
Additives	Xo, Mtb, Pva, Nano Gels	Crosslinker Antioxidant	Hydrophobic Dye, Organic Halogen, Tetrazolium Salts/Iodine	Dye, Halogen	Gold Nanoparticles, Mpy, Tbua, Nanoclay, Rd 123, Dhr 123, Halogen, Fe^3+^ Pyridine, Nanoclay, Gelatine, Agarose
Readout	OCT, MRI, UV-Vis	OCT, MRI, Raman	OCT, XCT, Raman	OCT, Raman	Optical

tBuA: Poly(tert-butyl acrylate; MPy: N-(1-pyrenyl)maleimide.

## Data Availability

No new data were created or analyzed in this study. Data sharing is not applicable to this article.
